# Comparison of Adverse Events Associated With Six Common Antibiotics in Lower Respiratory Tract Infections Using the United States Food and Drug Administration Adverse Event Reporting System

**DOI:** 10.7759/cureus.103809

**Published:** 2026-02-17

**Authors:** Toru Ogura, Chihiro Shiraishi

**Affiliations:** 1 Clinical Research Support Center, Mie University Hospital, Tsu, JPN; 2 Department of Emergency and Disaster Medical Pharmacy, Faculty of Pharmaceutical Sciences, Fukuoka University, Fukuoka, JPN

**Keywords:** antibiotic selection, clinical decision-making, database, pharmacovigilance, safety profile

## Abstract

Background

Lower respiratory tract infections (LRTIs) remain a major global health concern, contributing substantially to morbidity and mortality. Antibiotics are commonly prescribed for these infections, but their safety profiles may vary across drug classes and patient populations. Understanding patterns of reported adverse events (AEs) among patients with LRTIs could provide useful insights for pharmacovigilance.

Objectives

To explore and compare the safety profiles of six antibiotics frequently used for bacterial LRTIs using data from a spontaneous reporting database.

Methods

Data were obtained from the United States Food and Drug Administration Adverse Event Reporting System (FAERS) to identify AEs associated with these antibiotics in cases referring to LRTIs. Given the limitations of spontaneous reporting systems (e.g., underreporting, reporting bias, absence of denominators), this study was designed as hypothesis-generating. Time to AE onset was determined by the interval between antibiotic initiation and AE occurrence for cases with complete date information. Reporting odds ratios (RORs) and adjusted RORs (aRORs) were calculated to compare reporting proportions among antibiotics, with each antibiotic sequentially used as the reference in pairwise comparisons. Adjusted analyses accounted for sex, age, and reporter country, and Bonferroni correction adjusted the significance threshold to 0.0033.

Results

Across 11,522 patients, a total of 664 serious AEs were identified. Ciprofloxacin exhibited the highest reporting proportion, with 89 of 809 patients (11.0%), followed by clarithromycin (129/1,572; 8.2%), azithromycin (132/2,048; 6.4%), amoxicillin (90/1,796; 5.0%), levofloxacin (198/4,604; 4.3%), and doxycycline (26/693; 3.8%). Ciprofloxacin and clarithromycin were associated with a higher frequency of serious AEs compared with amoxicillin. For non‑serious AEs, dermatological disorders were reported less frequently with azithromycin, doxycycline, clarithromycin, levofloxacin, and ciprofloxacin than with amoxicillin, whereas neurological disorders were more frequently reported for doxycycline, clarithromycin, levofloxacin, and ciprofloxacin. Dermatological and lung disorders tended to appear early, typically within the first day after treatment initiation, whereas liver and renal disorders developed later (median ≥ 2 days). No single antibiotic exhibited consistently lower reporting proportions across all AE categories.

Conclusions

This FAERS‑based analysis identified antibiotic‑specific differences in both overall and serious AE reporting among commonly used agents for LRTIs. Fluoroquinolones (levofloxacin, ciprofloxacin) and macrolides (clarithromycin, azithromycin) showed elevated serious AE reporting relative to amoxicillin, underscoring the need for careful benefit-risk assessment. These drug‑specific patterns and temporal characteristics may help inform targeted clinical monitoring and guide future pharmacoepidemiological research to verify these hypothesis‑generating findings.

## Introduction

Lower respiratory tract infections (LRTIs) represent one of the most significant global health burdens [1]. These infections include conditions, such as pneumonia, bronchitis, and bronchiolitis, which can result in severe complications and death [1]. LRTIs are caused by a variety of pathogens, including viruses and bacteria [2]. Viral pathogens (such as influenza viruses, respiratory syncytial virus, and coronaviruses) are common causes of LRTIs, particularly in children and immunocompromised individuals. Bacterial pathogens, including *Streptococcus pneumoniae*, *Haemophilus influenzae*, and atypical organisms such as *Mycoplasma pneumoniae*, also contribute substantially, especially in adults and hospitalized patients. In 2019, LRTIs were responsible for approximately 2.4 million deaths worldwide, ranking as the third leading cause of death [1]. In addition, millions of hospitalizations each year are attributed to LRTIs. The management of LRTIs depends largely on the underlying etiology. While viral LRTIs are generally managed with supportive care, bacterial LRTIs frequently necessitate antibiotic therapy. Despite their indispensable role in treating bacterial LRTIs, inappropriate or excessive antibiotic use remains a recognized healthcare concern worldwide [3]. Such patterns of use have been linked not only to the rise of antimicrobial resistance but also to increased risks of antibiotic‑associated adverse events (AEs), which may complicate clinical management. Evaluating and comparing the safety profiles of commonly prescribed antibiotics is therefore important for informing rational antibiotic use and minimizing drug‑related risks. Six antibiotics are commonly prescribed for bacterial LRTIs: amoxicillin, azithromycin, doxycycline, clarithromycin, levofloxacin, and ciprofloxacin [4]. These agents target bacterial pathogens through distinct mechanisms of action [5]. Amoxicillin, a beta-lactam antibiotic, inhibits bacterial cell wall synthesis by binding to penicillin-binding proteins, leading to cell lysis, and is particularly effective against *Streptococcus pneumoniae*, a major cause of community-acquired pneumonia. Azithromycin and clarithromycin, both macrolides, inhibit bacterial protein synthesis by binding to the 50S ribosomal subunit and are especially active against atypical pathogens such as *Mycoplasma pneumoniae* and *Legionella *species. Doxycycline, a tetracycline antibiotic, also inhibits protein synthesis but targets the 30S ribosomal subunit, providing broad-spectrum activity against typical and atypical respiratory pathogens. Levofloxacin and ciprofloxacin, fluoroquinolone antibiotics, inhibit bacterial deoxyribonucleic acid (DNA) gyrase and topoisomerase IV (enzymes essential for DNA replication) and are particularly effective against Gram-negative pathogens such as Pseudomonas aeruginosa, which can cause severe LRTIs in hospitalized patients.

These differing mechanisms of action may lead to variations in efficacy and safety profiles. This highlights the need for comparative evaluations of AE profiles to better characterize the safety of these antibiotics in clinical practice. Although previous clinical trials have compared antibiotics, they have typically been restricted to head-to-head comparisons of one or two agents or comparisons with placebo [6,7]. Conducting clinical trials that simultaneously compare all six antibiotics would require an impractically large sample size, making such studies unfeasible within conventional clinical trial frameworks.

The United States Food and Drug Administration Adverse Event Reporting System (FAERS) [8] has been used in prior research to compare AE profiles among antibiotics. However, many of these studies lack specificity in patient populations and focus on a limited set of AEs, such as seizures [9] or acute kidney injury [10]. Such approaches may not fully account for the diverse indications of antibiotics or the potential differences in AE profiles across patient groups. In routine clinical practice, antibiotic choice is informed by a broad consideration of potential AEs rather than a single outcome. Consequently, existing FAERS-based studies provide only a partial view of safety and may not fully reflect real-world decision-making.

To address these gaps, the present study utilizes FAERS data to explore and compare the AE reporting profiles of six antibiotics specifically in the context of LRTI-related reports. By focusing on LRTI-specific cases and categorizing AEs into multiple organ systems and general health categories, this study aims to provide a more comprehensive, hypothesis-generating overview of the relative safety of these commonly used antibiotics. Rather than establishing causal relationships or incidence rates, our analysis seeks to identify patterns and temporal characteristics of reported AEs that may generate safety signals and complement evidence from clinical and epidemiological studies.

## Materials and methods

Data source

This study utilized the FAERS database, a publicly accessible repository of anonymized AE reports that has been updated quarterly since 2004. The FAERS database evolved from the Adverse Event Reporting System (AERS) in the fourth quarter of 2004 (2004Q1) to a more comprehensive format in 2012Q4. Data files from the AERS and FAERS, designated as aers_ascii_yyyyQq.zip and faers_ascii_yyyyQq.zip, where yyyy represents the year and q indicates the quarter, were accessed from the official FAERS website on January 31, 2025. For this study, we used data between 2004Q1 and 2025Q4. The data files selected for analysis included patient demographic and administrative information (DEMOyyQq.txt), drug information (DRUGyyQq.txt), AE information (REACyyQq.txt), drug therapy start and end dates (THERyyQq.txt), and indications for use (INDIyyQq.txt), where yy represents the last two digits of the year. These files provided comprehensive information necessary for analyzing drug-event associations within the context of LRTIs. To ensure consistency across databases, differences in variable names and structures between AERS and FAERS were reconciled based on their respective documentation. For instance, {ISR} in AERS was mapped to {primaryid} in FAERS for report identification, while {CASE} was mapped to {caseid} for case identification. Throughout this manuscript, curly braces are used to denote variable names as they appear in the FAERS database. The FAERS system uses a version control mechanism whereby updates to reports increment the version number {caseversion}, ensuring that previous data is not overwritten. For this study, only the highest {caseversion} number was used for each report to ensure data accuracy. In AERS, where {caseversion} was unavailable, equivalent cases were identified using unique report identifiers {ISR} and case numbers {CASE}.

Several preprocessing steps were undertaken to standardize and clean the data to ensure its reliability for analysis. The unit of the {age} variable was converted to years, and the unit of the {weight} variable was standardized to kilograms. Inconsistent or unexpected inputs for variables such as {sex}, {age}, {weight}, and the reporter's country {reporter_country} were systematically addressed to maintain data integrity. Additionally, missing line breaks in certain AERS files were manually inserted at specific lines (322,967 in DRUG11Q2.txt, 247,896 in DRUG11Q3.txt, and 446,738 in DRUG11Q4.txt) to ensure proper data parsing. Without these corrections, critical drug-event associations could have been misrepresented or excluded due to structural errors in the dataset. Importantly, these preprocessing steps did not alter the content of the data but restored its intended structure to ensure accurate parsing and reliable analysis.

Institutional review board approval was not required for this study because FAERS is a publicly accessible database containing anonymized data without linkable personal identifiers.

Study design

This study was designed to analyze AEs associated with six commonly used antibiotics (amoxicillin, azithromycin, doxycycline, clarithromycin, levofloxacin, and ciprofloxacin) prescribed for LRTIs. To ensure transparency and reproducibility, inclusion and exclusion criteria were meticulously defined based on specific FAERS variables and search terms.

The inclusion criteria targeted patients who received antibiotics specifically for LRTIs during the study period. For AERS data, cases were identified using both generic and brand names of antibiotics (listed in Appendix A) in the {drugname} (name of medicinal product) field, while for FAERS data, the {prod_ai} (product active ingredient) variable was employed to locate antibiotics by their generic names. Records were included only if the {indi_pt} (preferred term - level medical terminology describing the indication for use, using the Medical Dictionary for Regulatory Activities (MedDRA)) field explicitly indicated LRTIs, including pneumonia, bronchitis, and bronchiolitis. The specific indications (LRTIs, including pneumonia, bronchitis, and bronchiolitis) provided in the FAERS database that were considered for inclusion are detailed in Appendix B. To minimize confounding effects, cases involving upper respiratory tract infections were excluded from the analysis.

Exclusion criteria encompassed several conditions to enhance data reliability. Patients who initiated antibiotic therapy after the occurrence of AEs were excluded based on comparisons of {start_dt} (date the therapy was started or re-started for this drug), {event_dt} (date the AE occurred or began), and {end_dt} (date therapy was stopped for this drug). Records with missing date fields were retained to align with FAERS’s primary purpose of reporting AEs related to drug administration. Patients who received multiple antibiotics were excluded to avoid confounding effects from combination therapy. Additionally, cases where antibiotics were not identified as the primary suspect drug for the AE (determined using a variable indicating the drug's reported role in the event {role_cod}) were excluded. Duplicate entries were removed by identifying records with identical patient information across multiple variables (including {sex}, {age}, {weight}, {reporter_country}, {drugname}, {pt}, {start_dt}, {event_dt}, and {end_dt}) but differing identification numbers. This step ensured data integrity by eliminating redundant records.

The primary endpoint was the occurrence of AEs (described using variable of preferred term-level medical terminology describing the event, using MedDRA {pt}), categorized into 13 classifications: dermatological disorders, liver disorders, lung disorders, renal disorders, gastrointestinal disorders, neurological disorders, psychological disorders, musculoskeletal disorders, metabolic disorders, general fatigue disorders, circulatory disorders, hematological disorders, and serious AEs. Detailed descriptions of these categories are provided in Appendix C.

Statistical analyses

Continuous variables were summarized as medians with first and third quartiles, while categorical variables were summarized as frequencies with reporting proportions (RPs) [11]. The RP was calculated as (the number of patients reported with the category of interest) / (total number of patients reported receiving a particular antibiotic) × 100. The number of days from antibiotic administration to the onset of AEs was determined by subtracting the {start_dt} variable from the {event_dt} variable. Given that antibiotic-related AEs often occur within a few days of therapy initiation, only cases where both {start_dt} and {event_dt} were recorded with complete date information (year, month, and day) were included in analyses specifically examining this time to onset. For other statistical analyses that did not depend on this time interval, cases were included regardless of whether the exact number of days was available. To compare AE profiles among antibiotics, both reporting odds ratios (RORs) [12] and adjusted RORs (aRORs) were utilized. The ROR was calculated using univariate binomial logistic regression analysis, while the aROR was derived through multivariate binomial logistic regression analysis adjusted for sex, age, and reporter country. The {weight} variable was excluded from aROR calculations due to the high RP of unknown data. The {reporter_country} variable was treated as binary data (United States vs. other countries), as the United States contributed the majority of reports. Reference categories for adjustment variables were set as female for {sex} and other countries for {reporter_country}. Patients with unknown values for any adjustment variables were excluded from aROR calculations to maintain data integrity. Although imputation methods could have been employed, the limited number of patient background variables available in FAERS made accurate imputation impractical; thus, exclusion was deemed preferable. Pairwise comparisons among antibiotics were conducted by sequentially setting each antibiotic as the reference category, resulting in 15 comparisons overall. To account for multiple comparisons, the Bonferroni correction was applied to adjust the significance level to 0.05 divided by 15, yielding a threshold of 0.0033. Consequently, p < 0.0033 was considered statistically significant. The corresponding confidence interval (CI) was set at 99.67%, ensuring consistency between hypothesis testing and CI interpretation [13].

It is important to highlight that FAERS data consist solely of reported AEs and do not include information on cases where no AEs occurred. This limitation precludes calculation of true incidence rates for AEs, distinguishing FAERS-based studies from observational cohort studies or clinical trials that provide denominator data for incidence rate calculations. While cohort studies and clinical trials offer precise incidence rates based on controlled populations and study designs, FAERS captures real-world AE reporting across diverse populations but is subject to inherent biases such as underreporting and selective reporting. To emphasize this distinction from conventional statistical methods, "reporting" was prefixed to all statistical measures employed in this study, consistent with established practices in previous studies utilizing FAERS data. All statistical analyses were performed using R version 4.4.1 (R Foundation for Statistical Computing, Vienna, Austria).

## Results

Patient background

**Figure 1:**
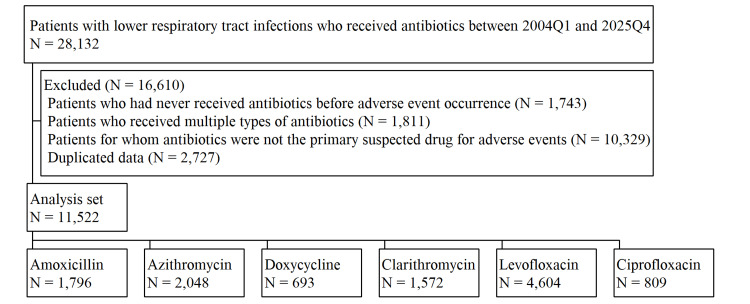
Flowchart of patients with lower respiratory tract infections who received antibiotics

Analysis of FAERS data between 2004Q1 and 2025Q4 identified a total of 28,132 patients who received antibiotics for LRTIs. Following the application of exclusion criteria, 16,610 patients were removed, resulting in a final cohort of 11,522 patients. The distribution of these patients across the six antibiotics is illustrated in Figure 1.

Specifically, amoxicillin was administered to 1,796 patients, azithromycin to 2,048 patients, doxycycline to 693 patients, clarithromycin to 1,572 patients, levofloxacin to 4,604 patients, and ciprofloxacin to 809 patients. A detailed summary of patient background variables is presented in Table 1. Across all antibiotics, female patients consistently outnumbered male patients. Median ages were relatively consistent across most antibiotics, ranging from 59 to 65 years. However, azithromycin exhibited notable deviations; its median age was lower at 51 years, with the first quartile at just 16 years, compared to at least 39 years for other antibiotics. This pattern reflects a higher proportion of pediatric patients in the azithromycin group. Similarly, median weights across most antibiotics ranged from 69 to 75 kg, but were lower for azithromycin at 60 kg. Geographically, the United States demonstrated the highest RP for azithromycin, levofloxacin, and ciprofloxacin among the six antibiotics analyzed. Previous studies have highlighted that macrolides such as azithromycin are commonly prescribed in the United States due to their broad-spectrum activity and convenience of short-course therapy, particularly for respiratory infections [14]. Similarly, fluoroquinolones like levofloxacin and ciprofloxacin are frequently utilized in the United States for their efficacy against a wide range of bacterial pathogens, including those causing complicated infections [15]. In contrast, doxycycline and clarithromycin exhibited higher RPs in the United Kingdom. These regional differences may reflect variations in national guidelines or regulatory influences favoring certain antibiotics for specific indications. For instance, doxycycline's elevated RP in the United Kingdom could be attributed to its widespread use for respiratory infections as recommended by local clinical guidelines [16]. The substantial variation in demographic and geographic distributions highlights the diverse patient populations using these antibiotics and underscores potential inherent biases in FAERS data reporting.

**Table 1:** Summary of patient characteristics Age and weight are summarized as median with first and third quartiles. Other variables are summarized as frequency (reporting proportion). As multiple indications could be documented for a single drug, the total count of indications may exceed the number of patients. Q1: first quartile; Q3: third quartile

Characteristics	Amoxicillin	Azithromycin	Doxycycline	Clarithromycin	Levofloxacin	Ciprofloxacin
	N = 1,796	N = 2,048	N = 693	N = 1,572	N = 4,604	N = 809
Sex, n (%)						
Female	923 (51.4)	1,067 (52.1)	427 (61.6)	827 (52.6)	2,638 (57.3)	426 (52.7)
Male	761 (42.4)	871 (42.5)	213 (30.7)	624 (39.7)	1,630 (35.4)	351 (43.4)
Unknown	112 (6.2)	110 (5.4)	53 (7.6)	121 (7.7)	336 (7.3)	32 (4.0)
Age, years						
Median	62.0	51.0	61.0	59.0	63.0	65.0
Q1–Q3	39.0–74.0	16.0–70.0	43.0–74.0	42.0 - 74.0	50.0 - 76.0	53.0–76.0
Unknown, n (%)	214 (11.9)	265 (12.9)	89 (12.8)	233 (14.8)	772 (16.8)	79 (9.8)
Weight, kg						
Median	70.0	60.0	75.0	69.6	73.5	70.0
Q1–Q3	57.1–84.0	35.2–77.1	61.1–88.9	56.8 - 81.0	62.2 - 88.4	56.0–84.8
Unknown, n (%)	1,096 (61.0)	1,076 (52.5)	351 (50.6)	892 (56.7)	1,975 (42.9)	469 (58.0)
Country, n (%)						
Canada	73 (4.1)	71 (3.5)	47 (6.8)	84 (5.3)	95 (2.1)	42 (5.2)
United States	208 (11.6)	822 (40.1)	183 (26.4)	157 (10.0)	2,860 (62.1)	183 (22.6)
China	1 (0.1)	329 (16.1)	7 (1.0)	5 (0.3)	10 (0.2)	10 (1.2)
Japan	4 (0.2)	134 (6.5)	2 (0.3)	107 (6.8)	294 (6.4)	60 (7.4)
France	462 (25.7)	114 (5.6)	2 (0.3)	44 (2.8)	70 (1.5)	48 (5.9)
Germany	69 (3.8)	49 (2.4)	13 (1.9)	49 (3.1)	105 (2.3)	48 (5.9)
Italy	89 (5.0)	59 (2.9)	3 (0.4)	112 (7.1)	285 (6.2)	32 (4.0)
Spain	71 (4.0)	65 (3.2)	0 (0.0)	20 (1.3)	167 (3.6)	14 (1.7)
United Kingdom	397 (22.1)	43 (2.1)	377 (54.4)	626 (39.8)	262 (5.7)	169 (20.9)
Others	367 (20.4)	259 (12.6)	47 (6.8)	249 (15.8)	194 (4.2)	144 (17.8)
Unknown	55 (3.1)	103 (5.0)	12 (1.7)	119 (7.6)	262 (5.7)	59 (7.3)
Indication category, n (%)						
Lower respiratory tract infection	403 (22.4)	79 (3.9)	357 (51.5)	565 (35.9)	189 (4.1)	143 (17.7)
Pneumonia	687 (38.3)	1,212 (59.2)	230 (33.2)	574 (36.5)	2,852 (61.9)	459 (56.7)
Bronchitis	700 (39.0)	700 (34.2)	106 (15.3)	430 (27.4)	1,602 (34.8)	208 (25.7)
Bronchiolitis	6 (0.3)	61 (3.0)	0 (0.0)	6 (0.4)	12 (0.3)	1 (0.1)

Adverse events

Table 2 provides a comprehensive summary of the AE categories associated with each antibiotic, illustrating notable variations in RPs across different AE categories. The analysis indicates that the types of AEs with high RPs vary depending on the specific antibiotic. For example, gastrointestinal disorders exhibited relatively high RPs for most antibiotics, while neurological and musculoskeletal disorders were more frequently associated with fluoroquinolones such as levofloxacin and ciprofloxacin.

**Table 2:** Summary of adverse event categories Data are summarized as frequency (reporting proportion). If multiple adverse events are reported in a patient, each adverse event is counted. Each patient is counted only once per adverse event category, regardless of the number of preferred terms experienced within that category.

Adverse event category	Amoxicillin	Azithromycin	Doxycycline	Clarithromycin	Levofloxacin	Ciprofloxacin
	N = 1,796	N = 2,048	N = 693	N = 1,572	N = 4,604	N = 809
Dermatological disorders	474 (26.4)	289 (14.1)	79 (11.4)	150 (9.5)	385 (8.4)	61 (7.5)
Liver disorders	167 (9.3)	87 (4.2)	40 (5.8)	74 (4.7)	65 (1.4)	35 (4.3)
Lung disorders	235 (13.1)	252 (12.3)	82 (11.8)	200 (12.7)	388 (8.4)	107 (13.2)
Renal disorders	62 (3.5)	27 (1.3)	26 (3.8)	55 (3.5)	53 (1.2)	32 (4.0)
Gastrointestinal disorders	303 (16.9)	336 (16.4)	153 (22.1)	310 (19.7)	460 (10.0)	89 (11.0)
Neurological disorders	129 (7.2)	206 (10.1)	117 (16.9)	389 (24.7)	947 (20.6)	130 (16.1)
Psychological disorders	23 (1.3)	42 (2.1)	26 (3.8)	123 (7.8)	321 (7.0)	48 (5.9)
Musculoskeletal disorders	57 (3.2)	90 (4.4)	40 (5.8)	110 (7.0)	2,068 (44.9)	186 (23.0)
Metabolic disorders	42 (2.3)	56 (2.7)	28 (4.0)	73 (4.6)	133 (2.9)	37 (4.6)
Circulatory disorders	115 (6.4)	164 (8.0)	41 (5.9)	127 (8.1)	293 (6.4)	52 (6.4)
General fatigue disorders	168 (9.4)	157 (7.7)	70 (10.1)	168 (10.7)	496 (10.8)	97 (12.0)
Hematological disorders	18 (1.0)	12 (0.6)	6 (0.9)	31 (2.0)	30 (0.7)	5 (0.6)
Serious adverse events	90 (5.0)	132 (6.4)	26 (3.8)	129 (8.2)	198 (4.3)	89 (11.0)

Table 3 summarizes the intervals between antibiotic administration and the onset of AEs, revealing distinct temporal patterns across AE categories. Dermatological and lung disorders tended to occur rapidly, with a median time to onset of ≤1 day for all antibiotics except ciprofloxacin, which showed a median of three days. These results suggest that close monitoring for these AEs is particularly important immediately after antibiotic initiation. In contrast, liver and renal disorders exhibited longer median times to onset (≥2 days) for all antibiotics, indicating that vigilance may be warranted several days after starting treatment. Furthermore, amoxicillin showed relatively delayed median times to onset for certain AE categories, such as renal and hematological disorders, compared with the other five antibiotics. These findings highlight potential differences in the temporal pattern of AE onset across antibiotics used and underscore the need to tailor monitoring strategies accordingly.

**Table 3:** Summary of time to onset of adverse events The percentage of patients used is calculated as (used n / adverse event category count from Table 2) × 100 for each cell. Q1: first quartile; Q3: third quartile; Used: number of patients included in the calculation of the Median (Q1–Q3)

Adverse event category	Median (Q1–Q3), days	Amoxicillin	Azithromycin	Doxycycline	Clarithromycin	Levofloxacin	Ciprofloxacin
Dermatological disorders	Median (Q1–Q3)	0.0 (0.0-3.0)	0.0 (0.0-2.0)	1.0 (0.0-3.0)	1.0 (0.0-3.0)	0.0 (0.0-3.0)	3.0 (0.0-7.5)
	Used, n (%)	316 (66.7)	235 (81.3)	57 (72.2)	113 (75.3)	244 (63.4)	35 (57.4)
Liver disorders	Median (Q1–Q3)	6.0 (2.5-14.0)	5.0 (2.8-8.5)	4.0 (2.0-7.0)	4.5 (2.0-7.8)	2.0 (0.0-7.2)	3.5 (1.0-5.2)
	Used, n (%)	103 (61.7)	64 (73.6)	22 (55.0)	38 (51.4)	36 (55.4)	16 (45.7)
Lung disorders	Median (Q1–Q3)	0.0 (0.0-2.0)	1.0 (0.0-3.0)	1.0 (0.0-3.0)	1.0 (0.0-4.0)	1.0 (0.0-6.0)	3.0 (1.0-6.5)
	Used, n (%)	113 (48.1)	153 (60.7)	49 (59.8)	125 (62.5)	244 (62.9)	43 (40.2)
Renal disorders	Median (Q1–Q3)	8.5 (2.8-18.0)	2.0 (1.0-3.0)	2.0 (1.0-5.0)	2.0 (0.0-6.0)	4.5 (2.0-8.0)	4.5 (2.8-8.0)
	Used, n (%)	36 (58.1)	13 (48.1)	12 (46.2)	25 (45.5)	30 (56.6)	12 (37.5)
Gastrointestinal disorders	Median (Q1–Q3)	2.0 (0.0-7.0)	0.0 (0.0-2.0)	1.0 (0.0-2.0)	1.0 (0.0-2.0)	2.0 (0.0-6.0)	3.0 (1.0-10.0)
	Used, n (%)	172 (56.8)	232 (69.0)	95 (62.1)	204 (65.8)	291 (63.3)	32 (36.0)
Neurological disorders	Median (Q1–Q3)	1.0 (0.0-3.0)	1.0 (0.0-2.0)	0.0 (0.0-1.0)	1.0 (0.0-3.0)	2.0 (0.0-5.0)	3.0 (1.0-7.0)
	Used, n (%)	81 (62.8)	139 (67.5)	81 (69.2)	298 (76.6)	662 (69.9)	55 (42.3)
Psychological disorders	Median (Q1–Q3)	1.0 (0.0-1.8)	2.0 (0.0-4.0)	1.0 (0.0-2.0)	2.0 (0.0-5.0)	2.0 (0.0-6.0)	3.0 (0.8-5.8)
	Used, n (%)	14 (60.9)	31 (73.8)	18 (69.2)	84 (68.3)	209 (65.1)	20 (41.7)
Musculoskeletal disorders	Median (Q1–Q3)	2.0 (0.0-6.0)	1.0 (0.0-2.5)	1.0 (0.0-3.5)	1.0 (0.0-4.0)	3.0 (0.0-8.0)	6.0 (2.0-23.0)
	Used, n (%)	33 (57.9)	55 (61.1)	23 (57.5)	72 (65.5)	1,397 (67.6)	91 (48.9)
Metabolic disorders	Median (Q1–Q3)	3.5 (2.0-9.5)	0.0 (0.0-1.0)	1.0 (1.0-3.0)	1.0 (0.0-2.0)	1.0 (0.0-4.0)	1.0 (1.0-5.2)
	Used, n (%)	26 (61.9)	41 (73.2)	13 (46.4)	49 (67.1)	83 (62.4)	20 (54.1)
Circulatory disorders	Median (Q1–Q3)	0.0 (0.0-2.0)	1.0 (0.0-3.0)	0.0 (0.0-1.0)	1.0 (0.0-3.0)	1.0 (0.0-5.0)	4.5 (1.0-14.5)
	Used, n (%)	71 (61.7)	109 (66.5)	20 (48.8)	79 (62.2)	185 (63.1)	22 (42.3)
General fatigue disorders	Median (Q1–Q3)	2.0 (0.0-9.5)	1.0 (0.0-3.0)	0.0 (0.0-2.0)	1.0 (0.0-3.0)	2.0 (0.0-6.8)	3.5 (0.2-23.0)
	Used, n (%)	80 (47.6)	98 (62.4)	36 (51.4)	122 (72.6)	342 (69.0)	38 (39.2)
Hematological disorders	Median (Q1–Q3)	6.0 (2.0-6.0)	3.0 (1.8-4.8)	0.0 (0.0-7.0)	2.0 (2.0-4.0)	2.0 (1.0-17.0)	1.0 (1.0-5.5)
	Used, n (%)	9 (50.0)	8 (66.7)	5 (83.3)	22 (71.0)	17 (56.7)	3 (60.0)
Serious adverse events	Median (Q1–Q3)	3.0 (1.0-13.0)	2.0 (0.0-7.2)	2.0 (2.0-3.2)	6.0 (1.0-11.0)	3.0 (1.0-8.0)	4.0 (1.0-8.5)
	Used, n (%)	37 (41.1)	58 (43.9)	8 (30.8)	69 (53.5)	83 (41.9)	43 (48.3)

Table 4 presents the ROR and aROR values for AE categories, using amoxicillin as the reference. Although aROR is generally preferred because it accounts for confounding factors, its calculation was limited by substantial unknown values in the FAERS database. For ROR, the sample sizes were 11,522 for antibiotic type, 10,758 for {sex}, 9,870 for {age}, and 10,912 for {reporter_country}. The sample size used for aROR was 9,233. Consequently, patients with unknown values in any of these variables were excluded from aROR calculations. This substantial reduction in sample size may introduce bias into the aROR estimates. To ensure robust findings, this study considered an AE category significant only when both ROR and aROR met the Bonferroni-adjusted significance threshold (p < 0.0033). This conservative approach minimizes false positives and enhances confidence in the identified associations. Given that ROR and aROR retain their inherent odds ratio properties [17], detailed results using different antibiotics as the reference can be derived directly from Table 4 [18]. Previous studies have provided R code for performing these calculations, enhancing the reproducibility and transparency of the analysis [12].

**Table 4:** ROR and aROR by adverse event category For ROR and aROR, the reference for antibiotic type, sex, and country were set to amoxicillin, female, and other countries, respectively. The sample sizes used for ROR were 11,522 for antibiotic type, 10,758 for sex, 9,870 for age, and 10,912 for country. The sample size employed for aROR remained consistent at 9,233. The Bonferroni correction was applied to adjust for multiple comparisons, setting the corrected significance threshold at 0.05 / 15 = 0.0033. aROR: adjusted reporting odds ratio; CI: confidence interval; ROR: reporting odds ratio

Adverse event category	ROR (99.67%CI)	p-value	aROR (99.67%CI)	p-value
Dermatological disorders				
Azithromycin	0.458 (0.359–0.585)	<0.0001	0.420 (0.318–0.555)	<0.0001
Doxycycline	0.359 (0.244–0.527)	<0.0001	0.414 (0.276–0.623)	<0.0001
Clarithromycin	0.294 (0.219–0.396)	<0.0001	0.278 (0.199–0.388)	<0.0001
Levofloxacin	0.255 (0.204–0.318)	<0.0001	0.306 (0.231–0.404)	<0.0001
Ciprofloxacin	0.227 (0.149–0.347)	<0.0001	0.276 (0.177–0.430)	<0.0001
Male	0.952 (0.801–1.133)	0.4091	0.893 (0.738–1.081)	0.0832
Age	0.986 (0.983–0.990)	<0.0001	0.988 (0.984–0.991)	<0.0001
United States	0.610 (0.508–0.732)	<0.0001	0.774 (0.615–0.973)	0.0010
Liver disorders				
Azithromycin	0.433 (0.290–0.646)	<0.0001	0.634 (0.406–0.991)	0.0028
Doxycycline	0.598 (0.350–1.019)	0.0047	0.587 (0.315–1.094)	0.0120
Clarithromycin	0.482 (0.316–0.736)	<0.0001	0.415 (0.251–0.686)	<0.0001
Levofloxacin	0.140 (0.090–0.216)	<0.0001	0.192 (0.113–0.328)	<0.0001
Ciprofloxacin	0.441 (0.252–0.773)	<0.0001	0.442 (0.238–0.820)	0.0001
Male	1.639 (1.228–2.188)	<0.0001	1.500 (1.094–2.056)	0.0002
Age	1.007 (1.000–1.014)	0.0016	1.009 (1.002–1.016)	0.0001
United States	0.266 (0.180–0.393)	<0.0001	0.470 (0.299–0.738)	<0.0001
Lung disorders				
Azithromycin	0.932 (0.701–1.239)	0.4683	0.820 (0.588–1.143)	0.0798
Doxycycline	0.891 (0.597–1.332)	0.4012	0.876 (0.561–1.368)	0.3827
Clarithromycin	0.968 (0.716–1.310)	0.7547	0.953 (0.678–1.341)	0.6808
Levofloxacin	0.611 (0.472–0.791)	<0.0001	0.578 (0.418–0.798)	<0.0001
Ciprofloxacin	1.012 (0.701–1.462)	0.9211	1.041 (0.694–1.562)	0.7720
Male	0.992 (0.825–1.192)	0.8952	0.952 (0.776–1.169)	0.4846
Age	0.997 (0.993–1.001)	0.0429	0.998 (0.994–1.002)	0.1748
United States	0.789 (0.653–0.953)	0.0002	1.043 (0.821–1.324)	0.6061
Renal disorders				
Azithromycin	0.374 (0.189–0.740)	<0.0001	0.551 (0.263–1.154)	0.0179
Doxycycline	1.090 (0.542–2.193)	0.7168	0.925 (0.408–2.098)	0.7793
Clarithromycin	1.014 (0.583–1.763)	0.9413	1.012 (0.548–1.870)	0.9531
Levofloxacin	0.326 (0.187–0.568)	<0.0001	0.378 (0.194–0.735)	<0.0001
Ciprofloxacin	1.152 (0.600–2.209)	0.5241	1.107 (0.548–2.235)	0.6715
Male	1.240 (0.839–1.833)	0.1054	1.221 (0.808–1.846)	0.1562
Age	1.020 (1.010–1.031)	<0.0001	1.021 (1.010–1.032)	<0.0001
United States	0.382 (0.240–0.609)	<0.0001	0.674 (0.387–1.173)	0.0365
Gastrointestinal disorders				
Azithromycin	0.967 (0.750–1.248)	0.6995	0.916 (0.680–1.235)	0.3884
Doxycycline	1.396 (1.007–1.935)	0.0027	1.519 (1.057–2.182)	0.0007
Clarithromycin	1.210 (0.931–1.573)	0.0327	1.381 (1.026–1.860)	0.0014
Levofloxacin	0.547 (0.433–0.692)	<0.0001	0.560 (0.416–0.753)	<0.0001
Ciprofloxacin	0.609 (0.417–0.889)	0.0001	0.697 (0.458–1.061)	0.0117
Male	0.626 (0.527–0.743)	<0.0001	0.595 (0.492–0.719)	<0.0001
Age	0.993 (0.989–0.996)	<0.0001	0.994 (0.990–0.998)	<0.0001
United States	0.934 (0.793–1.101)	0.2229	1.192 (0.965–1.472)	0.0146
Neurological disorders				
Azithromycin	1.445 (1.024–2.039)	0.0017	1.198 (0.806–1.780)	0.1807
Doxycycline	2.625 (1.758–3.919)	<0.0001	2.593 (1.663–4.041)	<0.0001
Clarithromycin	4.249 (3.090–5.842)	<0.0001	4.839 (3.378–6.932)	<0.0001
Levofloxacin	3.346 (2.507–4.467)	<0.0001	2.706 (1.909–3.837)	<0.0001
Ciprofloxacin	2.474 (1.678–3.649)	<0.0001	2.589 (1.674–4.002)	<0.0001
Male	0.550 (0.467–0.648)	<0.0001	0.568 (0.473–0.681)	<0.0001
Age	0.997 (0.994–1.001)	0.0298	0.995 (0.991–0.999)	0.0003
United States	1.674 (1.439–1.948)	<0.0001	1.687 (1.382–2.059)	<0.0001
Psychological disorders				
Azithromycin	1.614 (0.749–3.477)	0.0671	1.023 (0.428–2.448)	0.9386
Doxycycline	3.005 (1.283–7.035)	0.0001	2.888 (1.117–7.469)	0.0011
Clarithromycin	6.544 (3.332–12.850)	<0.0001	6.956 (3.288–14.713)	<0.0001
Levofloxacin	5.777 (3.050–10.946)	<0.0001	5.089 (2.412–10.735)	<0.0001
Ciprofloxacin	4.862 (2.285–10.347)	<0.0001	5.551 (2.395–12.862)	<0.0001
Male	0.745 (0.569–0.976)	0.0014	0.817 (0.603–1.108)	0.0516
Age	0.987 (0.982–0.992)	<0.0001	0.980 (0.973–0.986)	<0.0001
United States	1.845 (1.421–2.397)	<0.0001	1.654 (1.168–2.342)	<0.0001
Musculoskeletal disorders				
Azithromycin	1.402 (0.845–2.326)	0.0499	0.890 (0.510–1.555)	0.5411
Doxycycline	1.869 (1.005–3.475)	0.0031	1.300 (0.654–2.584)	0.2630
Clarithromycin	2.295 (1.406–3.748)	<0.0001	2.165 (1.262–3.715)	<0.0001
Levofloxacin	24.879 (16.601–37.284)	<0.0001	14.959 (9.560–23.407)	<0.0001
Ciprofloxacin	9.109 (5.721–14.501)	<0.0001	8.203 (4.947–13.602)	<0.0001
Male	0.635 (0.551–0.733)	<0.0001	0.729 (0.610–0.872)	<0.0001
Age	1.006 (1.003–1.009)	<0.0001	0.998 (0.993–1.002)	0.1227
United States	5.183 (4.476–6.002)	<0.0001	2.864 (2.363–3.471)	<0.0001
Metabolic disorders				
Azithromycin	1.174 (0.640–2.154)	0.4377	1.228 (0.608–2.479)	0.3918
Doxycycline	1.758 (0.849–3.643)	0.0230	2.102 (0.956–4.623)	0.0056
Clarithromycin	2.034 (1.141–3.624)	0.0003	2.043 (1.055–3.958)	0.0015
Levofloxacin	1.242 (0.734–2.102)	0.2261	1.419 (0.749–2.686)	0.1078
Ciprofloxacin	2.002 (1.020–3.926)	0.0025	2.000 (0.932–4.291)	0.0077
Male	1.078 (0.779–1.493)	0.4954	0.992 (0.694–1.419)	0.9497
Age	1.004 (0.996–1.011)	0.1485	1.002 (0.994–1.010)	0.4350
United States	0.945 (0.680–1.313)	0.6141	0.947 (0.626–1.434)	0.7025
Circulatory disorders				
Azithromycin	1.272 (0.879–1.843)	0.0562	1.331 (0.871–2.033)	0.0477
Doxycycline	0.919 (0.530–1.594)	0.6534	0.839 (0.451–1.562)	0.4080
Clarithromycin	1.285 (0.868–1.902)	0.0608	1.348 (0.871–2.084)	0.0447
Levofloxacin	0.993 (0.712–1.387)	0.9541	0.951 (0.632–1.429)	0.7165
Ciprofloxacin	1.004 (0.605–1.667)	0.9811	0.988 (0.564–1.730)	0.9493
Male	0.842 (0.668–1.061)	0.0287	0.811 (0.629–1.044)	0.0149
Age	1.004 (0.999–1.010)	0.0136	1.005 (1.000–1.011)	0.0067
United States	0.950 (0.755–1.195)	0.5109	1.011 (0.758–1.347)	0.9131
General fatigue disorders				
Azithromycin	0.805 (0.572–1.131)	0.0609	0.814 (0.549–1.208)	0.1259
Doxycycline	1.089 (0.701–1.690)	0.5702	1.086 (0.660–1.787)	0.6260
Clarithromycin	1.160 (0.827–1.625)	0.1981	1.237 (0.838–1.825)	0.1089
Levofloxacin	1.170 (0.888–1.542)	0.0946	1.191 (0.840–1.687)	0.1415
Ciprofloxacin	1.320 (0.888–1.963)	0.0399	1.547 (0.995–2.405)	0.0037
Male	0.721 (0.592–0.879)	<0.0001	0.724 (0.583–0.899)	<0.0001
Age	0.998 (0.994–1.002)	0.1319	0.997 (0.992–1.001)	0.0337
United States	1.224 (1.014–1.478)	0.0016	1.243 (0.976–1.582)	0.0082
Hematological disorders				
Azithromycin	0.582 (0.194–1.745)	0.1481	0.676 (0.181–2.527)	0.3839
Doxycycline	0.863 (0.215–3.463)	0.7551	1.077 (0.257–4.512)	0.8790
Clarithromycin	1.987 (0.828–4.770)	0.0214	1.942 (0.727–5.191)	0.0475
Levofloxacin	0.648 (0.269–1.560)	0.1471	0.769 (0.264–2.241)	0.4718
Ciprofloxacin	0.614 (0.139–2.722)	0.3367	0.580 (0.110–3.057)	0.3365
Male	0.731 (0.381–1.402)	0.1573	0.635 (0.310–1.299)	0.0624
Age	1.022 (1.005–1.040)	0.0001	1.022 (1.004–1.040)	0.0003
United States	0.488 (0.244–0.977)	0.0024	0.603 (0.247–1.475)	0.0970
Serious adverse events				
Azithromycin	1.306 (0.864-1.974)	0.0578	1.640 (1.022-2.632)	0.0022
Doxycycline	0.739 (0.379-1.440)	0.1829	0.825 (0.391-1.738)	0.4480
Clarithromycin	1.695 (1.117-2.570)	0.0002	1.794 (1.121-2.870)	0.0003
Levofloxacin	0.852 (0.581-1.249)	0.2184	1.001 (0.628-1.594)	0.9969
Ciprofloxacin	2.343 (1.483-3.703)	<0.0001	2.069 (1.224-3.497)	<0.0001
Male	1.940 (1.519-2.478)	<0.0001	1.824 (1.388-2.398)	<0.0001
Age	1.010 (1.004-1.016)	<0.0001	1.009 (1.003-1.015)	<0.0001
United States	0.559 (0.424-0.735)	<0.0001	0.662 (0.474-0.926)	0.0003

Across 11,522 patients, a total of 664 serious AEs were identified. Ciprofloxacin exhibited the highest RP, with 89 of 809 patients (11.0%), followed by clarithromycin (129/1,572; 8.2%), azithromycin (132/2,048; 6.4%), amoxicillin (90/1,796; 5.0%), levofloxacin (198/4,604; 4.3%), and doxycycline (26/693; 3.8%). When compared with amoxicillin, ciprofloxacin and azithromycin were associated with significantly higher reporting of serious AEs (ciprofloxacin: ROR 2.343 {99.67% CI: 1.483-3.703}, p < 0.0002; aROR 2.069 {99.67% CI: 1.224-3.497}, p < 0.0001), and clarithromycin likewise showed increased reporting (ROR 1.695 {99.67% CI: 1.117-2.570}, p = 0.0002; aROR 1.794 {99.67% CI: 1.121-2.870}, p = 0.0003). The median time to onset of serious AEs ranged from two to six days across antibiotics, indicating that such events generally emerged within the first week of therapy. With regard to non‑serious AE categories, only statistically significant findings are summarized here. Relative to amoxicillin, dermatological disorders were reported significantly less frequently (ROR < 1, aROR < 1) with azithromycin, doxycycline, clarithromycin, levofloxacin, and ciprofloxacin. Similarly, liver disorders were reported less frequently with azithromycin, clarithromycin, levofloxacin, and ciprofloxacin, and lung and renal disorders were less frequently reported with levofloxacin. In contrast, neurological and psychological disorders were reported more frequently (ROR > 1, aROR > 1) with doxycycline, clarithromycin, levofloxacin, and ciprofloxacin, with comparable patterns observed for musculoskeletal and metabolic disorders, depending on the antibiotic.

When other antibiotics were used as references, variations in AE reporting patterns were observed across categories. No single antibiotic consistently showed lower reporting proportions across all AEs, indicating that each agent exhibits a distinct reporting profile. These findings suggest potential differences in AE reporting among antibiotics and highlight the importance of interpreting such results cautiously in conjunction with clinical and pharmacoepidemiological evidence.

## Discussion

The treatment of LRTIs has evolved significantly, driven by the development of diverse antibiotic classes with distinct mechanisms of action. This diversification has expanded therapeutic options, but it also necessitates a detailed understanding of the safety profiles associated with each antibiotic. Our study provides a comprehensive evaluation of AEs linked to six widely used antibiotics in the context of LRTI management.

Levofloxacin demonstrated a significantly higher RP for musculoskeletal disorders compared to the other five antibiotics analyzed. Previous studies have consistently reported an increased risk of tendinitis and tendon rupture associated with fluoroquinolones, particularly levofloxacin, which may explain the elevated RP observed in this study [19]. Experimental studies have shown that fluoroquinolones, including levofloxacin, induce direct cytotoxic effects on tendon cells, increase matrix-degrading proteolytic activity, and reduce collagen synthesis within tendon tissues, leading to structural weakening and increased susceptibility to rupture.. Clinical and epidemiological investigations have further documented fluoroquinolone-related musculoskeletal complications, with levofloxacin being among the agents most frequently implicated [20,21]. These preclinical and clinical findings are consistent with our FAERS results showing significantly higher reporting proportions for musculoskeletal disorders with levofloxacin than with other antibiotics (Table 4). Moreover, real-world pharmacovigilance data, including recent analyses focusing on pediatric populations, have similarly highlighted musculoskeletal safety signals for fluoroquinolones [22]. The widespread distribution of levofloxacin in musculoskeletal tissues, including tendons, muscles, and connective tissues, may further potentiate these effects [23].

Amoxicillin demonstrated a notably higher RP for dermatological disorders compared to the other antibiotics analyzed in this study. This finding is consistent with previous research, which has identified beta-lactam antibiotics, including amoxicillin, as a leading cause of antibiotic-induced skin reactions [24]. Studies have shown that amoxicillin is frequently associated with cutaneous AEs, such as maculopapular rashes, urticaria, and more severe conditions like Stevens-Johnson syndrome and toxic epidermal necrolysis.

Clarithromycin and levofloxacin demonstrated notably high RPs for neurological disorders in this study. These findings align with previous research that highlights the neurotoxic potential of both antibiotics. Clarithromycin, a macrolide antibiotic, has been associated with central nervous system side effects such as dizziness, headaches, delirium, and hallucinations [25]. Studies suggest that clarithromycin-induced neurotoxicity may be linked to its inhibition of cytochrome P450 enzymes, particularly CYP3A4, which can lead to elevated drug concentrations in susceptible individuals. Levofloxacin, a fluoroquinolone antibiotic, is similarly associated with a range of neurological side effects, including insomnia, restlessness, seizures, and encephalopathy. The neurotoxicity of fluoroquinolones is thought to involve the inhibition of gamma-aminobutyric acid receptors and activation of N-methyl-D-aspartate receptors in the CNS. In our LRTI-focused FAERS analysis, serious AEs were reported more frequently for ciprofloxacin and clarithromycin than for amoxicillin, though reporting frequencies do not reflect incidence. Pharmacoepidemiologic work on prolonged antibiotic exposure found that severe AEs requiring hospitalization did not show remarkably large risk increases with long-term ciprofloxacin, doxycycline, or amoxicillin use, but still highlighted the importance of monitoring for clinically relevant serious events [26]. These complementary findings suggest that ciprofloxacin and certain broader-spectrum agents warrant particular vigilance for serious AEs, even if the overall absolute risk may vary by population and exposure duration.

Higher RPs in female patients were observed across all antibiotics, which may be due to various reasons. This pattern aligns with prior research, including the systematic review, which demonstrated that females are prescribed antibiotics more frequently than men in community settings, even after accounting for conditions like urinary tract infections [27]. Similarly, adult females received approximately twice as many antibiotic prescriptions as men in English primary care, primarily driven by higher consultation rates among females [28]. It should be noted that physiological changes that occur throughout women's lives, such as menstruation, pregnancy, and menopause, increase susceptibility to infections and necessitate the use of antibiotics [29].

This study has several limitations. The FAERS database, being a voluntary reporting system, is inherently subject to biases such as underreporting and selective reporting, particularly for mild or transient AEs. Additionally, FAERS lacks denominator data on the total number of patients exposed to each antibiotic, which precludes the calculation of true incidence rates for AEs. The absence of key patient variables, such as weight, further limited our ability to fully adjust for confounding factors in the aROR analyses. These limitations highlight the challenges of interpreting data from pharmacovigilance systems like FAERS.

The strengths of this study are its focused approach and rigorous methodology. By limiting the analysis to LRTI cases, we enhanced the specificity and clinical relevance of our findings. Additionally, the use of FAERS data allowed us to capture real-world AE reporting across diverse populations, providing valuable insights into antibiotic-associated risks in heterogeneous clinical settings. The application of both ROR and aROR, along with the conservative requirement for statistical significance in both measures, further bolsters the reliability of our results. This dual-significance approach reduces false positives and ensures robust identification of associations between antibiotics and specific AEs. The Bonferroni correction applied to account for multiple comparisons adds statistical rigor to our analysis.

Despite these inherent constraints, the FAERS database offers significant advantages, particularly its large volume of globally collected reports. Conducting clinical trials to simultaneously compare all six antibiotics would require an impractically large sample size, making such studies unfeasible within traditional research frameworks. However, the broad FAERS database allows for analyses that are challenging to implement with other databases. The large sample size allowed for adequately powered statistical analyses, even with explicit inclusion and exclusion criteria and the application of Bonferroni corrections to account for multiple comparisons. These methodological approaches minimized biases and confounding factors inherent in FAERS data and facilitated a more robust analysis of AE profiles across the six antibiotics studied. The application of rigorous selection criteria and advanced statistical methods helped mitigate some of the limitations associated with FAERS data. These measures enhanced the reliability of the findings while leveraging the strengths of a large-scale pharmacovigilance database. Nevertheless, it is essential to emphasize that results derived from FAERS should be regarded as hypothesis-generating rather than definitive. Further validation through controlled studies, including observational cohort studies or randomized clinical trials, is necessary to confirm these findings and provide deeper insights into the associations observed. By utilizing FAERS data in this manner, this study underscores the potential of pharmacovigilance databases to address research questions that are impractical to explore through traditional clinical trial designs.

## Conclusions

FAERS analysis revealed distinct AE reporting profiles among six antibiotics for lower respiratory tract infections, with fluoroquinolones showing elevated neurological and musculoskeletal signals and amoxicillin associated with higher dermatological reports. Dermatological and lung disorders manifested rapidly after treatment initiation, while liver and renal disorders appeared later across agents. Serious AEs were more frequently reported with ciprofloxacin and clarithromycin than with amoxicillin, underscoring the need for careful benefit-risk assessment when these agents are selected for LRTIs. These agent-specific patterns and temporal characteristics inform targeted clinical monitoring strategies-early vigilance for skin and pulmonary risks, delayed assessment for hepatic and renal complications-enhancing patient safety within existing healthcare systems. Pharmacovigilance databases like FAERS complement clinical trials by efficiently generating multi-agent safety hypotheses unattainable through conventional study designs, and future pharmacoepidemiologic research should validate these signals and quantify clinical risks, including serious AEs, in defined LRTI populations.

## Appendices

Appendix A

**Table 5:** Generic and brand names of six antibiotics

Generic name	Brand name
Amoxicillin	Amoxi, Amoxil, Moxatag
Azithromycin	Z-Pak, Zithromax, Zmax
Doxycycline	Adoxa, Doryx, Monodox, Vibramycin
Clarithromycin	Biaxin, Klaricid
Levofloxacin	Cravit, Levaquin, Tavanic
Ciprofloxacin	Ciloxan, Cipro, Proquin

Appendix B

**Table 6:** Classification of lower respiratory tract infections from indications provided in the FAERS database FAERS: United States Food and Drug Administration Adverse Event Reporting System

Indication category	Specific indications
Lower respiratory tract infection	Lower respiratory tract infection
Pneumonia	Bronchopneumonia, Eosinophilic pneumonia, Lobar pneumonia, Organising pneumonia, Pneumonia, Pneumonia aspiration, Pneumonia bacterial, Pneumonia chlamydial, Pneumonia fungal, Pneumonia klebsiella, Pneumonia legionella, Pneumonia lipoid, Pneumonia mycoplasmal, Pneumonia pneumococcal, Pneumonia pseudomonal, Pneumonia staphylococcal, Pneumonia streptococcal
Bronchitis	Bronchitis, Bronchitis acute, Bronchitis bacterial, Bronchitis chronic, Bronchitis mycoplasmal, Chronic bronchitis
Bronchiolitis	Bronchiolitis, Bronchiolitis obliterans syndrome, Diffuse panbronchiolitis, Obliterative bronchiolitis

Appendix C

**Table 7:** Categorization of preferred terms

Adverse event category	Preferred terms
Dermatological disorders	Anaphylactic reaction, Anaphylactic shock, Angioedema, Dermatitis allergic, Erythema, Hypersensitivity, Lip swelling, Rash, Swelling face, Urticaria
Liver disorders	Alanine aminotransferase increased, Drug-induced liver injury, Gamma-glutamyltransferase increased, Hepatic enzyme increased, Hepatitis, Jaundice, Liver disorder, Liver function test abnormal, Liver function test increased, Liver injury
Lung disorders	Asthma, Chest pain, Cough, Dyspnoea, Electrocardiogram qt prolonged, Hemoptysis, Oxygen saturation decreased, Pneumonia, Wheezing
Renal disorders	Acute kidney injury, Chromaturia, Renal impairment
Gastrointestinal disorders	Abdominal discomfort, Abdominal distension, Abdominal pain, Abdominal pain lower, Abdominal pain upper, Diarrhoea, Dysphagia, Gastrointestinal hemorrhage, Gastrooesophageal reflux disease, Nausea, Vomiting
Neurological disorders	Confusional state, Disorientation, Disturbance in attention, Dizziness, Dysgeusia, Hallucination, Headache, Hypoesthesia, Insomnia, Lethargy, Loss of consciousness, Paresthesia, Vision blurred
Psychological disorders	Agitation, Anxiety, Depression, Psychotic disorder, Suicidal ideation
Musculoskeletal disorders	Arthralgia, Gait disturbance, Joint swelling, Muscle spasms, Muscular weakness, Musculoskeletal pain, Musculoskeletal stiffness, Myalgia, Pain in extremity, Tendon pain, Tendon rupture, Tendonitis
Metabolic disorders	Dehydration, Hyperhidrosis, Hypoglycemia
General fatigue disorders	Asthenia, Fatigue, Malaise, Pyrexia
Circulatory disorders	Atrial fibrillation, Blood pressure increased, Blood pressure systolic increased, Cardiac arrest, Hypotension, Myocardial ischemia, Palpitations, Pulmonary embolism, Syncope, Tachycardia
Hematological disorders	Epistaxis, Hemorrhage, Melena
Serious adverse events	Acute hepatic failure, Arrhythmia, Cardiac failure, Death, Myocardial infarction, Renal failure, Renal failure acute, Respiratory failure, Rhabdomyolysis, Septic shock, Sepsis, Stevens-Johnson syndrome, Torsade de pointes, Ventricular fibrillation
